# Ca_4_As_3_ – a new binary calcium arsenide

**DOI:** 10.1107/S2056989015022367

**Published:** 2015-11-28

**Authors:** Andrea V. Hoffmann, Viktor Hlukhyy, Thomas F. Fässler

**Affiliations:** a Technische Universität München, Department of Chemistry, Lichtenbergstr. 4, 85747 Garching, Germany

**Keywords:** crystal structure, arsenide, Zintl phase

## Abstract

The crystal structure of the binary compound tetra­calcium triarsenide, Ca_4_As_3_, was investigated by single-crystal X-ray diffraction. Ca_4_As_3_ crystallizes in the Ba_4_P_3_ structure type and is thus a homologue of isotypic Sr_4_As_3_. The unit cell contains 32 Ca^2+^ cations, 16 As^3−^ isolated anions and four centrosymmetric [As_2_]^4–^ dumbbells. The As atoms in each of the dumbbells are connected by a single bond, thus this calcium arsenide is a Zintl phase.

## Abstract 2

The binary compound Ca_4_As_3_crystallizes in the Ba_4_P_3_ structure type and is thus a homologue of isotypic Sr_4_As_3_. The As atoms are connected by a single bond thus this calcium arsenide is a Zintl phase.

## Chemical context   

Six binary compounds have been reported so far in the binary phase diagram Ca–As: CaAs_3_ (Brice *et al.*, 1976 ▸), Ca_2_As_3_ (Deller & Eisenmann, 1976 ▸), CaAs (Iandelli & Franceschi, 1973 ▸), Ca_16_As_11_ (Leon-Escamilla *et al.*, 1997 ▸), Ca_5_As_3_ (Hütz & Nagorsen, 1975 ▸) and Ca_2_As (Hütz & Nagorsen, 1974 ▸). In the binary phase system, the following trend is observed: with increasing As-content the number of covalent As—As bonds increases. Ca_2_As and Ca_5_As_3_ are reported as inter­metallic phases. In the Ca-richest compound Ca_2_As, the Ca atoms in the first coordination sphere of As adopt a monocapped square-anti­prismatic geometry (CN = 9), while the Ca atoms are situated inside cubocta­hedra (eight Ca and four As atoms) or 13-vertex polyhedra (eight Ca and five As atoms). In Ca_5_As_3_, nine Ca atoms form deformed monocapped square anti­prisms around As while the coordination polyhedra of Ca atoms are formed by bicapped-hexa­gonal anti­prismatic (eight Ca and six As atoms) and 15-vertex polyhedra (ten Ca and five As atoms). No covalent bonds are found in either compound. The other four Ca–As compounds are Zintl phases containing polyanionic As-substructures. The polyarsenic substructure varies with the atomic percentage of Ca. Compounds with 50–59.3 at.% of Ca (CaAs, Ca_16_As_11_ and the title compound Ca_4_As_3_) all contain [As_2_]^4–^ dumbbells as a structure motif. Ca_2_As_3_ contains two types of As chains: [As_4_]^6–^ and [As_8_]^10–^. The structure of the CaAs_3_ compound with the highest As content contains a two-dimensional [As_3_]^2–^ network as a polyarsenic substructure besides the three bonded [As]^0^ and two bonded [As]^1–^ atoms in a ratio of 1:2.

## Structural commentary   

**Figure 1:**
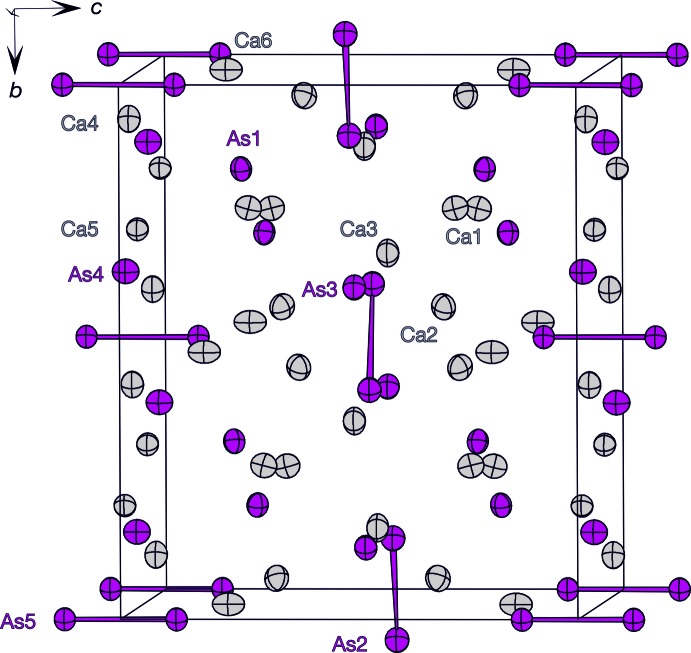
Crystal structure of Ca_4_As_3_ shown along the *a* axis. The Ca atoms are shown in gray and the As atoms in magenta as anisotropic displacement ellipsoids with a 90% probability level. The As—As dumbbell bonds are shown in magenta.

**Figure 2:**
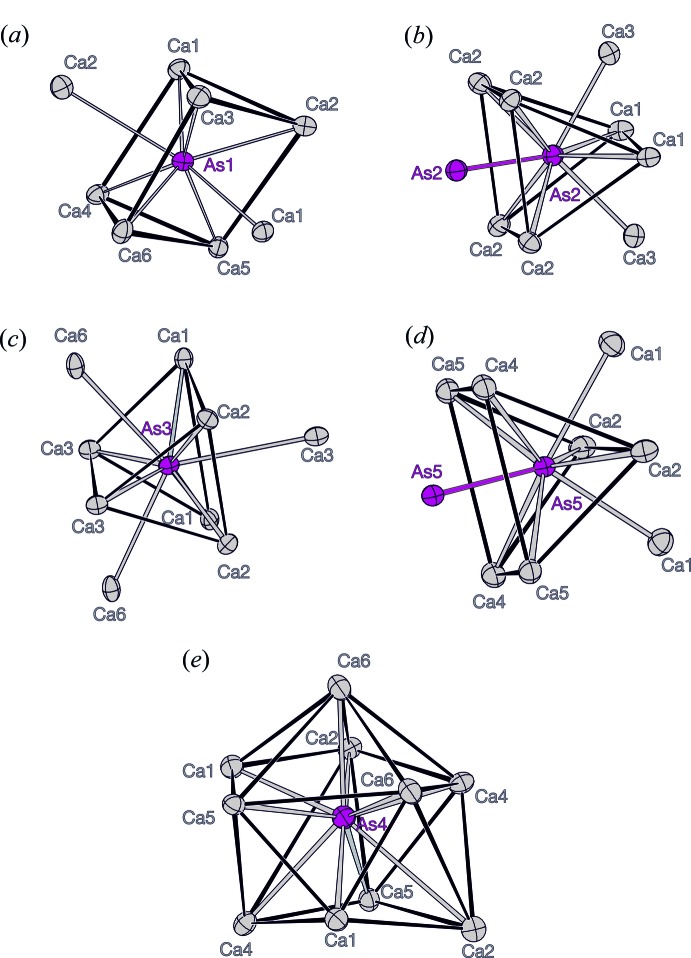
The coordination polyhedra of As atoms. The Ca atoms are shown in gray and the As atoms in magenta as anisotropic displacement ellipsoids with a 90% probability level. The As—As dumbbell bonds are emphasized in magenta.

The unit cell of the title compounds is shown in Fig. 1 ▸. The phase Ca_4_As_3_ (*Z* = 8) with 57 at.% Ca fulfils the 8-N rule according to a salt-like compound: the charge of 32 Ca^2+^ cations are counterbalanced by 16 isolated As^3–^ anions and four [As_2_]^4–^ dumbbells (two As2—As2 dumbbells and two As5—As5 dumbbells) per unit cell. The dumbbells formed by the As2 anions, with an inter­atomic distance of of 2.507 (2) Å, lie in the *ab* plane. The second type of dumbbells of the As5 anions, with *d*(As5—As5) of 2.527 (2) Å, lie along the *c*-axis direction, thus the two dumbbells are oriented perpendicular with respect to each other. Both As—As distances are in the range of covalent single bonds observed in elemental As and other binary Ca–As compounds (2.44–2.57 Å). Each As atom of the dumbbells is coordinated by eight Ca cations. Six Ca cations form a distorted trigonal prism while two Ca cations cap two of the rectangular faces of the prism; the third rectangular prism face is capped by the covalently bonded As atom (Fig. 2*b*,*d* ▸). The two trigonal prisms around As2 or As5 share their tetra­gonal faces, each with the As dumbbell in the center of the eight-vertex polyhedron of Ca atoms. Two of the As^3–^ anions (As1 and As3) that are not bonded to further As atoms are coordinated by Ca atoms in form of distorted trigonal prisms (Fig. 2*a*,*c* ▸). For As1, two faces of the prism are capped while for As3, three faces are capped with Ca atoms. The trigonal–prismatic coordination polyhedra of As1 and As3 are connected by sharing edges. In contrast to the other As atoms, As4 possesses a different coordination sphere having also the highest coordination number (CN = 10) of Ca atoms, forming a polyhedron with 14 faces (Fig. 2*e* ▸). The coordination sphere can be described as an icosa­hedron with two removed adjacent vertices.

**Figure 3:**
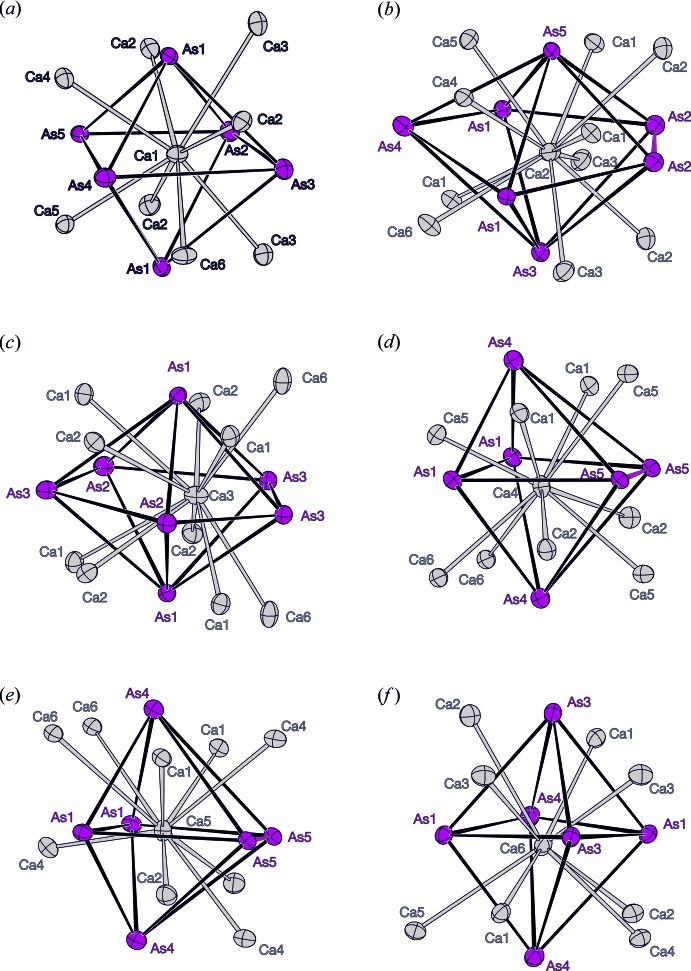
The coordination polyhedra of Ca atoms. The Ca atoms are shown in gray and the As atoms in magenta as anisotropic displacement ellipsoids with a 90% probability level. The As—As dumbbells are emphasized in magenta.

The coordination around the Ca cations is formed by six or seven As atoms and eight to ten Ca atoms (Fig. 3 ▸). Distorted octa­hedra are formed by six As atoms around Ca1, Ca4, Ca5 and Ca6. For Ca4 and Ca5, one edge is formed by an As5 dumbbell. The faces of the octa­hedra are capped by Ca atoms. In most cases *d*(Ca—Ca) is longer than 3.5 Å; however, a rather short distance of 3.289 (2) Å is observed between Ca4 and Ca5. Those two Ca atoms are coordinated by the As5 dumbbells (Fig. 3 ▸ *d*,*e*). The distorted As octa­hedra around Ca4 and Ca5 share a common face (As1–As1–As4) in the *ab* plane. Ca2 and Ca3 are surrounded by seven As atoms (Fig. 3 ▸ *b*,*c*). In both cases, the coordination polyhedron resembles a distorted penta­gonal bipyramid. For Ca2, one edge of the penta­gon is an As2 dumbbell. Each of the trigonal faces is capped by Ca atoms.

## Comparison with isostructural compounds   

Comparison of Ca_4_As_3_ with the isostructural Sr_4_As_3_ (Somer *et al.*, 1995 ▸) and Ba_4_P_3_ (Hadenfeldt *et al.*, 1993 ▸) show that the lattice parameters increase in accordance with the cation size. The distances in the As–As dumbbells for Sr_4_As_3_ are 2.52 and 2.55 Å, which is slightly longer than observed in dumbbells of Ca_4_As_3_ [2.507 (2) Å and 2.527 (2) Å, respectively]. The lattice parameters for Ba_4_P_3_ are further increased due to the larger Ba atoms. However, the distances in the dumbbells [*d*(P—P) of 2.25 and 2.32 Å] are shorter than in the As compounds due to the smaller covalent radius of P.

## Synthesis and crystallization   

Single crystals of the title compound were obtained from experiments aiming at an alloy with the nominal composition of 12Ca:10Fe:10As:4Rh:8Si. A mixture of Ca (2.35 mmol), Fe (1.96 mmol), As (1.96 mmol) and pre-prepared ‘Rh:2Si’ precursor (0.78 mmol) was placed in an alumina crucible which was sealed in a tantalum ampoule under an argon atmosphere. The ampoule was heated in a resistances furnace to 1373 K and held for 24 h. Afterwards, the temperature was reduced to 1248 K at a rate of 0.1 K min^−1^ and held there for a week. Single crystals of the title compound could be isolated from the product. Energy-dispersive X-ray analysis (EDX) of the crystals showed an atomic ratio of Ca/As close to 4:3 in all analysed crystals. No impurity elements heavier than sodium were observed. The binary Ca_4_As_3_ phase was subsequently synthesized from the pure elements.

## Refinement   

Crystal data, data collection and structure refinement details are summarized in Table 1 ▸. All atoms were refined with anisotropic displacement parameters. The remaining maximum and minimum electron densities are located 1.29 Å from As2 and 0.03 Å from As5, respectively.

## Supplementary Material

Crystal structure: contains datablock(s) global, I. DOI: 10.1107/S2056989015022367/ru2065sup1.cif

Structure factors: contains datablock(s) I. DOI: 10.1107/S2056989015022367/ru2065Isup2.hkl

Supporting information file. DOI: 10.1107/S2056989015022367/ru2065Isup3.cml

CCDC reference: 1438335

Additional supporting information:  crystallographic information; 3D view; checkCIF report

## Figures and Tables

**Table 1:** Experimental details

Crystal data	
Chemical formula	Ca_4_As_3_
*M*_r_	385.08
Crystal system, space group	Orthorhombic, *P* *b* *a* *m*
Temperature (K)	150
*a*, *b*, *c* (Å)	11.5137 (5), 12.0584 (6), 10.3426 (4)
*V* (Å^3^)	1435.93 (11)
*Z*	8
Radiation type	Mo *K*α
μ (mm^−1^)	16.61
Crystal size (mm)	0.08 × 0.04 × 0.01

Data collection	
Diffractometer	Oxford Diffraction Xcalibur 3
Absorption correction	Multi-scan (*CrysAlis RED*; Oxford Diffraction, 2009 ▸)
*T*_min_, *T*_max_	0.683, 1.000
No. of measured, independent and observed [*I* > 2σ(*I*)] reflections	26414, 2659, 1273
*R*_int_	0.143
(sin θ/λ)_max_ (Å^−1^)	0.764

Refinement	
*R*[*F* ^2^ > 2σ(*F* ^2^)], *wR*(*F* ^2^), *S*	0.034, 0.054, 0.68
No. of reflections	2659
No. of parameters	74
Δρ_max_, Δρ_min_ (e Å^−3^)	1.95, −1.56

Computer programs: *CrysAlis CCD* and *CrysAlis RED* (Oxford Diffraction, 2009 ▸), *SHELXS97* and *SHELXL97* (Sheldrick, 2008 ▸) and *DIAMOND* (Brandenburg, 2012 ▸).

## supplementary crystallographic information

### Crystal data

**Table:**

Ca_4_As_3_	*F*(000) = 1432
*M**_r_* = 385.08	*D*_x_ = 3.563 Mg m^−^^3^
Orthorhombic, *P**b**a**m*	Mo *K*α radiation, λ = 0.71073 Å
Hall symbol: -P 2 2ab	Cell parameters from 2659 reflections
*a* = 11.5137 (5) Å	θ = 3.4–32.9°
*b* = 12.0584 (6) Å	µ = 16.61 mm^−^^1^
*c* = 10.3426 (4) Å	*T* = 150 K
*V* = 1435.93 (11) Å^3^	Needle, metallic dark grey
*Z* = 8	0.08 × 0.04 × 0.01 mm

### Data collection

**Table:**

Oxford Diffraction Xcalibur 3 diffractometer	2659 independent reflections
Radiation source: Enhance (Mo) X-ray Source	1273 reflections with *I* > 2σ(*I*)
Graphite monochromator	*R*_int_ = 0.143
Detector resolution: 16.0238 pixels mm^-1^	θ_max_ = 32.9°, θ_min_ = 3.4°
ω and π scans	*h* = −17→17
Absorption correction: multi-scan (*CrysAlis RED*; Oxford Diffraction, 2009)	*k* = −18→18
*T*_min_ = 0.683, *T*_max_ = 1.000	*l* = −14→14
26414 measured reflections	

### Refinement

**Table:**

Refinement on *F*^2^	0 restraints
Least-squares matrix: full	Primary atom site location: structure-invariant direct methods
*R*[*F*^2^ > 2σ(*F*^2^)] = 0.034	Secondary atom site location: difference Fourier map
*wR*(*F*^2^) = 0.054	*w* = 1/[σ^2^(*F*_o_^2^) + (0.0138*P*)^2^] where *P* = (*F*_o_^2^ + 2*F*_c_^2^)/3
*S* = 0.68	(Δ/σ)_max_ < 0.001
2659 reflections	Δρ_max_ = 1.95 e Å^−^^3^
74 parameters	Δρ_min_ = −1.56 e Å^−^^3^

### Special details

**Table:**

Geometry. All e.s.d.'s (except the e.s.d. in the dihedral angle between two l.s. planes) are estimated using the full covariance matrix. The cell e.s.d.'s are taken into account individually in the estimation of e.s.d.'s in distances, angles and torsion angles; correlations between e.s.d.'s in cell parameters are only used when they are defined by crystal symmetry. An approximate (isotropic) treatment of cell e.s.d.'s is used for estimating e.s.d.'s involving l.s. planes.
Refinement. Refinement of *F*^2^ against ALL reflections. The weighted *R*-factor *wR* and goodness of fit *S* are based on *F*^2^, conventional *R*-factors *R* are based on *F*, with *F* set to zero for negative *F*^2^. The threshold expression of *F*^2^ > σ(*F*^2^) is used only for calculating *R*-factors(gt) *etc*. and is not relevant to the choice of reflections for refinement. *R*-factors based on *F*^2^ are statistically about twice as large as those based on *F*, and *R*- factors based on ALL data will be even larger.

### Fractional atomic coordinates and isotropic or equivalent isotropic displacement parameters (Å^2^)

**Table:**

	*x*	*y*	*z*	*U*_iso_*/*U*_eq_	
As1	0.33718 (5)	0.17628 (5)	0.23336 (5)	0.00964 (12)	
As2	0.03879 (7)	0.09712 (7)	0.5000	0.01132 (19)	
As3	0.13520 (7)	0.38636 (7)	0.5000	0.01069 (19)	
As4	0.14021 (7)	0.35690 (7)	0.0000	0.01187 (19)	
As5	0.0000	0.0000	0.12216 (8)	0.00906 (18)	
Ca1	0.09403 (10)	0.23535 (9)	0.27341 (10)	0.0113 (2)	
Ca2	0.32326 (10)	0.43298 (9)	0.32339 (10)	0.0122 (2)	
Ca3	0.37493 (14)	0.13596 (14)	0.5000	0.0134 (4)	
Ca4	0.22573 (13)	0.07676 (14)	0.0000	0.0110 (4)	
Ca5	0.39968 (14)	0.29309 (13)	0.0000	0.0103 (4)	
Ca6	0.0000	0.5000	0.18518 (16)	0.0129 (3)	

### Atomic displacement parameters (Å^2^)

**Table:**

	*U*^11^	*U*^22^	*U*^33^	*U*^12^	*U*^13^	*U*^23^
As1	0.0085 (3)	0.0117 (3)	0.0087 (3)	0.0010 (2)	−0.0005 (3)	0.0004 (2)
As2	0.0109 (4)	0.0121 (5)	0.0110 (5)	−0.0005 (3)	0.000	0.000
As3	0.0099 (4)	0.0122 (4)	0.0101 (4)	−0.0019 (3)	0.000	0.000
As4	0.0096 (4)	0.0118 (4)	0.0141 (4)	0.0002 (3)	0.000	0.000
As5	0.0079 (4)	0.0106 (4)	0.0087 (4)	0.0008 (3)	0.000	0.000
Ca1	0.0089 (5)	0.0118 (6)	0.0132 (6)	0.0003 (5)	0.0002 (5)	0.0002 (5)
Ca2	0.0119 (6)	0.0135 (6)	0.0112 (6)	0.0002 (5)	0.0023 (5)	−0.0009 (5)
Ca3	0.0148 (9)	0.0154 (9)	0.0099 (8)	−0.0018 (7)	0.000	0.000
Ca4	0.0091 (8)	0.0142 (9)	0.0098 (8)	−0.0018 (7)	0.000	0.000
Ca5	0.0119 (8)	0.0096 (8)	0.0096 (8)	−0.0016 (7)	0.000	0.000
Ca6	0.0081 (8)	0.0103 (8)	0.0202 (9)	0.0003 (7)	0.000	0.000

### Geometric parameters (Å, º)

**Table:**

As1—Ca3	2.8338 (6)	Ca2—As5^i^	3.0209 (12)
As1—Ca6^i^	2.8777 (6)	Ca2—As2^i^	3.1027 (13)
As1—Ca5	2.8857 (10)	Ca2—As2^viii^	3.1268 (13)
As1—Ca1	2.9182 (13)	Ca2—As1^xv^	3.5897 (13)
As1—Ca4	2.9853 (11)	Ca2—Ca2^iv^	3.653 (2)
As1—Ca1^i^	3.1707 (13)	Ca2—Ca1^i^	3.7559 (16)
As1—Ca2	3.2364 (13)	Ca2—Ca1^xv^	3.8037 (16)
As1—Ca2^ii^	3.5898 (13)	Ca2—Ca4^x^	3.8093 (14)
As2—As2^iii^	2.5068 (17)	Ca2—Ca3^viii^	3.8123 (18)
As2—Ca1	2.9453 (12)	Ca3—As1^iv^	2.8338 (6)
As2—Ca1^iv^	2.9453 (12)	Ca3—As3^i^	3.0088 (18)
As2—Ca2^v^	3.1026 (13)	Ca3—As3^vii^	3.0119 (18)
As2—Ca2^vi^	3.1026 (13)	Ca3—Ca1^xvi^	3.7768 (17)
As2—Ca2^ii^	3.1267 (13)	Ca3—Ca1^i^	3.7768 (17)
As2—Ca2^vii^	3.1267 (13)	Ca3—Ca2^ii^	3.8123 (18)
As3—Ca2	2.8880 (13)	Ca3—Ca2^vii^	3.8123 (18)
As3—Ca2^iv^	2.8880 (13)	Ca3—Ca6^i^	3.9196 (16)
As3—Ca1^iv^	3.0054 (12)	Ca3—Ca6^vii^	3.9196 (16)
As3—Ca1	3.0054 (12)	Ca3—Ca2^iv^	4.0643 (19)
As3—Ca3^v^	3.0088 (18)	Ca4—As1^xi^	2.9853 (11)
As3—Ca3^viii^	3.0119 (18)	Ca4—As5^xii^	3.0344 (14)
As4—Ca6	3.0417 (12)	Ca4—As4^xiii^	3.0677 (19)
As4—Ca6^ix^	3.0417 (12)	Ca4—Ca5	3.289 (2)
As4—Ca4^x^	3.0678 (19)	Ca4—Ca5^xiii^	3.713 (2)
As4—Ca5	3.0850 (18)	Ca4—Ca1^xi^	3.7353 (15)
As4—Ca1^xi^	3.2291 (12)	Ca4—Ca6^i^	3.8075 (16)
As4—Ca1	3.2291 (12)	Ca4—Ca6^xiii^	3.8075 (16)
As4—Ca5^v^	3.3076 (18)	Ca5—As1^xi^	2.8857 (10)
As4—Ca4	3.5186 (19)	Ca5—As5^i^	3.0258 (14)
As5—As5^xii^	2.5268 (16)	Ca5—As5^x^	3.0258 (14)
As5—Ca2^ii^	3.0209 (12)	Ca5—As4^i^	3.3076 (18)
As5—Ca2^v^	3.0209 (12)	Ca5—Ca1^i^	3.6223 (15)
As5—Ca5^xiii^	3.0258 (14)	Ca5—Ca1^xvii^	3.6223 (15)
As5—Ca5^v^	3.0258 (14)	Ca5—Ca4^x^	3.713 (2)
As5—Ca4^xii^	3.0344 (14)	Ca5—Ca2^xi^	3.8480 (13)
As5—Ca4	3.0344 (14)	Ca6—As1^xv^	2.8777 (6)
As5—Ca1	3.4166 (12)	Ca6—As1^v^	2.8777 (6)
As5—Ca1^xiv^	3.4166 (12)	Ca6—As4^ix^	3.0417 (12)
Ca1—As1^v^	3.1707 (13)	Ca6—Ca1^xviii^	3.4913 (12)
Ca1—Ca6	3.4913 (12)	Ca6—Ca4^v^	3.8075 (16)
Ca1—Ca2	3.5933 (16)	Ca6—Ca4^x^	3.8075 (16)
Ca1—Ca5^v^	3.6223 (15)	Ca6—Ca6^ix^	3.831 (3)
Ca1—Ca4	3.7353 (15)	Ca6—Ca3^viii^	3.9196 (16)
Ca1—Ca2^v^	3.7559 (16)	Ca6—Ca3^v^	3.9196 (16)
Ca1—Ca3^v^	3.7768 (17)		
			
Ca3—As1—Ca6^i^	86.67 (5)	As1—Ca3—As3^i^	99.68 (3)
Ca3—As1—Ca5	149.25 (5)	As1^iv^—Ca3—As3^vii^	99.53 (4)
Ca6^i^—As1—Ca5	93.05 (4)	As1—Ca3—As3^vii^	99.53 (4)
Ca3—As1—Ca1	92.92 (4)	As3^i^—Ca3—As3^vii^	87.09 (5)
Ca6^i^—As1—Ca1	146.08 (3)	As1^iv^—Ca3—Ca1^xvi^	55.12 (3)
Ca5—As1—Ca1	103.82 (4)	As1—Ca3—Ca1^xvi^	129.48 (5)
Ca3—As1—Ca4	141.60 (5)	As3^i^—Ca3—Ca1^xvi^	51.06 (3)
Ca6^i^—As1—Ca4	80.97 (4)	As3^vii^—Ca3—Ca1^xvi^	115.88 (4)
Ca5—As1—Ca4	68.11 (4)	As1^iv^—Ca3—Ca1^i^	129.48 (5)
Ca1—As1—Ca4	78.49 (4)	As1—Ca3—Ca1^i^	55.12 (3)
Ca3—As1—Ca1^i^	77.73 (4)	As3^i^—Ca3—Ca1^i^	51.06 (3)
Ca6^i^—As1—Ca1^i^	70.32 (3)	As3^vii^—Ca3—Ca1^i^	115.88 (4)
Ca5—As1—Ca1^i^	73.30 (4)	Ca1^xvi^—Ca3—Ca1^i^	76.70 (4)
Ca1—As1—Ca1^i^	142.58 (3)	As1^iv^—Ca3—Ca2^ii^	118.99 (5)
Ca4—As1—Ca1^i^	129.91 (4)	As1—Ca3—Ca2^ii^	63.39 (3)
Ca3—As1—Ca2	83.78 (4)	As3^i^—Ca3—Ca2^ii^	122.60 (5)
Ca6^i^—As1—Ca2	142.05 (3)	As3^vii^—Ca3—Ca2^ii^	48.34 (3)
Ca5—As1—Ca2	77.65 (4)	Ca1^xvi^—Ca3—Ca2^ii^	163.92 (5)
Ca1—As1—Ca2	71.23 (3)	Ca1^i^—Ca3—Ca2^ii^	111.49 (3)
Ca4—As1—Ca2	126.57 (4)	As1^iv^—Ca3—Ca2^vii^	63.39 (3)
Ca1^i^—As1—Ca2	71.77 (3)	As1—Ca3—Ca2^vii^	118.99 (5)
Ca3—As1—Ca2^ii^	71.72 (4)	As3^i^—Ca3—Ca2^vii^	122.60 (5)
Ca6^i^—As1—Ca2^ii^	77.08 (2)	As3^vii^—Ca3—Ca2^vii^	48.34 (3)
Ca5—As1—Ca2^ii^	138.09 (4)	Ca1^xvi^—Ca3—Ca2^vii^	111.49 (3)
Ca1—As1—Ca2^ii^	70.67 (3)	Ca1^i^—Ca3—Ca2^vii^	163.92 (5)
Ca4—As1—Ca2^ii^	70.12 (3)	Ca2^ii^—Ca3—Ca2^vii^	57.26 (4)
Ca1^i^—As1—Ca2^ii^	136.12 (3)	As1^iv^—Ca3—Ca6^i^	159.48 (6)
Ca2—As1—Ca2^ii^	132.97 (3)	As1—Ca3—Ca6^i^	47.13 (2)
As2^iii^—As2—Ca1	127.28 (3)	As3^i^—Ca3—Ca6^i^	66.21 (3)
As2^iii^—As2—Ca1^iv^	127.28 (3)	As3^vii^—Ca3—Ca6^i^	66.19 (3)
Ca1—As2—Ca1^iv^	105.44 (5)	Ca1^xvi^—Ca3—Ca6^i^	116.23 (4)
As2^iii^—As2—Ca2^v^	66.78 (3)	Ca1^i^—Ca3—Ca6^i^	53.91 (2)
Ca1—As2—Ca2^v^	76.73 (3)	Ca2^ii^—Ca3—Ca6^i^	63.47 (2)
Ca1^iv^—As2—Ca2^v^	135.02 (4)	Ca2^vii^—Ca3—Ca6^i^	110.45 (4)
As2^iii^—As2—Ca2^vi^	66.78 (3)	As1^iv^—Ca3—Ca6^vii^	47.13 (2)
Ca1—As2—Ca2^vi^	135.02 (4)	As1—Ca3—Ca6^vii^	159.48 (6)
Ca1^iv^—As2—Ca2^vi^	76.73 (3)	As3^i^—Ca3—Ca6^vii^	66.21 (3)
Ca2^v^—As2—Ca2^vi^	72.13 (4)	As3^vii^—Ca3—Ca6^vii^	66.19 (3)
As2^iii^—As2—Ca2^ii^	65.77 (4)	Ca1^xvi^—Ca3—Ca6^vii^	53.91 (2)
Ca1—As2—Ca2^ii^	77.51 (3)	Ca1^i^—Ca3—Ca6^vii^	116.23 (4)
Ca1^iv^—As2—Ca2^ii^	135.51 (4)	Ca2^ii^—Ca3—Ca6^vii^	110.45 (4)
Ca2^v^—As2—Ca2^ii^	89.33 (4)	Ca2^vii^—Ca3—Ca6^vii^	63.47 (2)
Ca2^vi^—As2—Ca2^ii^	132.54 (3)	Ca6^i^—Ca3—Ca6^vii^	112.34 (5)
As2^iii^—As2—Ca2^vii^	65.77 (4)	As1^iv^—Ca3—Ca2^iv^	52.34 (3)
Ca1—As2—Ca2^vii^	135.51 (4)	As1—Ca3—Ca2^iv^	105.29 (5)
Ca1^iv^—As2—Ca2^vii^	77.51 (3)	As3^i^—Ca3—Ca2^iv^	102.98 (4)
Ca2^v^—As2—Ca2^vii^	132.54 (3)	As3^vii^—Ca3—Ca2^iv^	151.04 (3)
Ca2^vi^—As2—Ca2^vii^	89.33 (4)	Ca1^xvi^—Ca3—Ca2^iv^	57.10 (3)
Ca2^ii^—As2—Ca2^vii^	71.49 (4)	Ca1^i^—Ca3—Ca2^iv^	90.83 (4)
Ca2—As3—Ca2^iv^	78.47 (5)	Ca2^ii^—Ca3—Ca2^iv^	133.91 (5)
Ca2—As3—Ca1^iv^	136.83 (4)	Ca2^vii^—Ca3—Ca2^iv^	105.24 (3)
Ca2^iv^—As3—Ca1^iv^	75.11 (3)	Ca6^i^—Ca3—Ca2^iv^	142.72 (4)
Ca2—As3—Ca1	75.11 (3)	Ca6^vii^—Ca3—Ca2^iv^	92.81 (2)
Ca2^iv^—As3—Ca1	136.83 (4)	As1^iv^—Ca3—Ca2	105.29 (5)
Ca1^iv^—As3—Ca1	102.48 (5)	As1—Ca3—Ca2	52.34 (3)
Ca2—As3—Ca3^v^	139.83 (3)	As3^i^—Ca3—Ca2	102.98 (4)
Ca2^iv^—As3—Ca3^v^	139.83 (3)	As3^vii^—Ca3—Ca2	151.03 (3)
Ca1^iv^—As3—Ca3^v^	77.80 (3)	Ca1^xvi^—Ca3—Ca2	90.83 (4)
Ca1—As3—Ca3^v^	77.80 (3)	Ca1^i^—Ca3—Ca2	57.10 (3)
Ca2—As3—Ca3^viii^	80.48 (4)	Ca2^ii^—Ca3—Ca2	105.24 (3)
Ca2^iv^—As3—Ca3^viii^	80.48 (4)	Ca2^vii^—Ca3—Ca2	133.91 (5)
Ca1^iv^—As3—Ca3^viii^	126.82 (3)	Ca6^i^—Ca3—Ca2	92.81 (2)
Ca1—As3—Ca3^viii^	126.82 (3)	Ca6^vii^—Ca3—Ca2	142.72 (4)
Ca3^v^—As3—Ca3^viii^	92.91 (5)	Ca2^iv^—Ca3—Ca2	53.41 (4)
Ca6—As4—Ca6^ix^	78.05 (5)	As1—Ca4—As1^xi^	107.90 (5)
Ca6—As4—Ca4^x^	77.10 (3)	As1—Ca4—As5^xii^	145.83 (4)
Ca6^ix^—As4—Ca4^x^	77.10 (3)	As1^xi^—Ca4—As5^xii^	98.87 (2)
Ca6—As4—Ca5	130.95 (3)	As1—Ca4—As5	98.87 (2)
Ca6^ix^—As4—Ca5	130.95 (3)	As1^xi^—Ca4—As5	145.83 (4)
Ca4^x^—As4—Ca5	74.23 (4)	As5^xii^—Ca4—As5	49.21 (4)
Ca6—As4—Ca1^xi^	136.18 (4)	As1—Ca4—As4^xiii^	97.54 (4)
Ca6^ix^—As4—Ca1^xi^	67.59 (3)	As1^xi^—Ca4—As4^xiii^	97.54 (4)
Ca4^x^—As4—Ca1^xi^	118.37 (2)	As5^xii^—Ca4—As4^xiii^	99.63 (5)
Ca5—As4—Ca1^xi^	92.65 (3)	As5—Ca4—As4^xiii^	99.63 (5)
Ca6—As4—Ca1	67.59 (3)	As1—Ca4—Ca5	54.51 (3)
Ca6^ix^—As4—Ca1	136.18 (4)	As1^xi^—Ca4—Ca5	54.51 (3)
Ca4^x^—As4—Ca1	118.37 (2)	As5^xii^—Ca4—Ca5	139.78 (5)
Ca5—As4—Ca1	92.65 (3)	As5—Ca4—Ca5	139.78 (5)
Ca1^xi^—As4—Ca1	122.26 (5)	As4^xiii^—Ca4—Ca5	112.28 (6)
Ca6—As4—Ca5^v^	82.29 (3)	As1—Ca4—As4	74.59 (4)
Ca6^ix^—As4—Ca5^v^	82.29 (3)	As1^xi^—Ca4—As4	74.60 (4)
Ca4^x^—As4—Ca5^v^	153.36 (5)	As5^xii^—Ca4—As4	93.05 (4)
Ca5—As4—Ca5^v^	132.41 (3)	As5—Ca4—As4	93.05 (4)
Ca1^xi^—As4—Ca5^v^	67.29 (3)	As4^xiii^—Ca4—As4	166.04 (5)
Ca1—As4—Ca5^v^	67.29 (3)	Ca5—Ca4—As4	53.77 (4)
Ca6—As4—Ca4	133.88 (3)	As1—Ca4—Ca5^xiii^	122.51 (3)
Ca6^ix^—As4—Ca4	133.88 (3)	As1^xi^—Ca4—Ca5^xiii^	122.51 (3)
Ca4^x^—As4—Ca4	133.54 (3)	As5^xii^—Ca4—Ca5^xiii^	52.11 (3)
Ca5—As4—Ca4	59.31 (4)	As5—Ca4—Ca5^xiii^	52.11 (3)
Ca1^xi^—As4—Ca4	67.07 (3)	As4^xiii^—Ca4—Ca5^xiii^	53.09 (4)
Ca1—As4—Ca4	67.07 (3)	Ca5—Ca4—Ca5^xiii^	165.37 (6)
Ca5^v^—As4—Ca4	73.10 (4)	As4—Ca4—Ca5^xiii^	140.86 (5)
As5^xii^—As5—Ca2^ii^	133.55 (2)	As1—Ca4—Ca1	49.96 (3)
As5^xii^—As5—Ca2^v^	133.55 (2)	As1^xi^—Ca4—Ca1	125.50 (6)
Ca2^ii^—As5—Ca2^v^	92.90 (5)	As5^xii^—Ca4—Ca1	97.10 (4)
As5^xii^—As5—Ca5^xiii^	65.320 (18)	As5—Ca4—Ca1	59.55 (3)
Ca2^ii^—As5—Ca5^xiii^	79.04 (3)	As4^xiii^—Ca4—Ca1	130.29 (3)
Ca2^v^—As5—Ca5^xiii^	139.94 (3)	Ca5—Ca4—Ca1	80.86 (4)
As5^xii^—As5—Ca5^v^	65.320 (18)	As4—Ca4—Ca1	52.76 (3)
Ca2^ii^—As5—Ca5^v^	139.94 (3)	Ca5^xiii^—Ca4—Ca1	108.29 (4)
Ca2^v^—As5—Ca5^v^	79.04 (3)	As1—Ca4—Ca1^xi^	125.50 (6)
Ca5^xiii^—As5—Ca5^v^	130.64 (4)	As1^xi^—Ca4—Ca1^xi^	49.96 (3)
As5^xii^—As5—Ca4^xii^	65.395 (18)	As5^xii^—Ca4—Ca1^xi^	59.55 (3)
Ca2^ii^—As5—Ca4^xii^	141.46 (4)	As5—Ca4—Ca1^xi^	97.10 (4)
Ca2^v^—As5—Ca4^xii^	77.97 (3)	As4^xiii^—Ca4—Ca1^xi^	130.29 (3)
Ca5^xiii^—As5—Ca4^xii^	84.35 (4)	Ca5—Ca4—Ca1^xi^	80.86 (4)
Ca5^v^—As5—Ca4^xii^	75.56 (4)	As4—Ca4—Ca1^xi^	52.76 (3)
As5^xii^—As5—Ca4	65.395 (18)	Ca5^xiii^—Ca4—Ca1^xi^	108.29 (4)
Ca2^ii^—As5—Ca4	77.97 (3)	Ca1—Ca4—Ca1^xi^	98.41 (5)
Ca2^v^—As5—Ca4	141.46 (4)	As1—Ca4—Ca6^i^	48.28 (3)
Ca5^xiii^—As5—Ca4	75.57 (4)	As1^xi^—Ca4—Ca6^i^	98.51 (4)
Ca5^v^—As5—Ca4	84.35 (4)	As5^xii^—Ca4—Ca6^i^	147.74 (6)
Ca4^xii^—As5—Ca4	130.79 (4)	As5—Ca4—Ca6^i^	115.26 (3)
As5^xii^—As5—Ca1	117.25 (2)	As4^xiii^—Ca4—Ca6^i^	51.14 (3)
Ca2^ii^—As5—Ca1	72.14 (3)	Ca5—Ca4—Ca6^i^	71.80 (4)
Ca2^v^—As5—Ca1	71.08 (3)	As4—Ca4—Ca6^i^	117.74 (4)
Ca5^xiii^—As5—Ca1	139.04 (4)	Ca5^xiii^—Ca4—Ca6^i^	95.66 (4)
Ca5^v^—As5—Ca1	68.11 (3)	Ca1—Ca4—Ca6^i^	94.61 (3)
Ca4^xii^—As5—Ca1	135.68 (4)	Ca1^xi^—Ca4—Ca6^i^	147.35 (5)
Ca4—As5—Ca1	70.48 (3)	As1—Ca4—Ca6^xiii^	98.51 (4)
As5^xii^—As5—Ca1^xiv^	117.25 (2)	As1^xi^—Ca4—Ca6^xiii^	48.28 (3)
Ca2^ii^—As5—Ca1^xiv^	71.08 (3)	As5^xii^—Ca4—Ca6^xiii^	115.26 (3)
Ca2^v^—As5—Ca1^xiv^	72.14 (3)	As5—Ca4—Ca6^xiii^	147.74 (6)
Ca5^xiii^—As5—Ca1^xiv^	68.11 (3)	As4^xiii^—Ca4—Ca6^xiii^	51.14 (3)
Ca5^v^—As5—Ca1^xiv^	139.04 (4)	Ca5—Ca4—Ca6^xiii^	71.80 (4)
Ca4^xii^—As5—Ca1^xiv^	70.48 (3)	As4—Ca4—Ca6^xiii^	117.74 (4)
Ca4—As5—Ca1^xiv^	135.68 (4)	Ca5^xiii^—Ca4—Ca6^xiii^	95.66 (4)
Ca1—As5—Ca1^xiv^	125.50 (4)	Ca1—Ca4—Ca6^xiii^	147.35 (5)
As1—Ca1—As2	100.48 (4)	Ca1^xi^—Ca4—Ca6^xiii^	94.61 (3)
As1—Ca1—As3	96.16 (4)	Ca6^i^—Ca4—Ca6^xiii^	60.40 (5)
As2—Ca1—As3	75.91 (3)	As1—Ca5—As1^xi^	113.52 (6)
As1—Ca1—As1^v^	163.39 (4)	As1—Ca5—As5^i^	98.54 (2)
As2—Ca1—As1^v^	95.32 (4)	As1^xi^—Ca5—As5^i^	147.88 (4)
As3—Ca1—As1^v^	92.60 (3)	As1—Ca5—As5^x^	147.88 (4)
As1—Ca1—As4	80.13 (3)	As1^xi^—Ca5—As5^x^	98.54 (2)
As2—Ca1—As4	171.57 (4)	As5^i^—Ca5—As5^x^	49.36 (4)
As3—Ca1—As4	112.45 (4)	As1—Ca5—As4	83.12 (4)
As1^v^—Ca1—As4	83.49 (3)	As1^xi^—Ca5—As4	83.12 (4)
As1—Ca1—As5	92.08 (3)	As5^i^—Ca5—As4	99.44 (4)
As2—Ca1—As5	79.97 (3)	As5^x^—Ca5—As4	99.44 (4)
As3—Ca1—As5	155.54 (4)	As1—Ca5—Ca4	57.38 (3)
As1^v^—Ca1—As5	85.63 (3)	As1^xi^—Ca5—Ca4	57.38 (3)
As4—Ca1—As5	91.62 (3)	As5^i^—Ca5—Ca4	152.44 (3)
As1—Ca1—Ca6	118.91 (4)	As5^x^—Ca5—Ca4	152.44 (3)
As2—Ca1—Ca6	131.17 (4)	As4—Ca5—Ca4	66.93 (5)
As3—Ca1—Ca6	72.48 (4)	As1—Ca5—As4^i^	86.67 (4)
As1^v^—Ca1—Ca6	50.906 (19)	As1^xi^—Ca5—As4^i^	86.67 (4)
As4—Ca1—Ca6	53.65 (3)	As5^i^—Ca5—As4^i^	97.54 (4)
As5—Ca1—Ca6	122.77 (4)	As5^x^—Ca5—As4^i^	97.54 (4)
As1—Ca1—Ca2	58.51 (3)	As4—Ca5—As4^i^	161.30 (6)
As2—Ca1—Ca2	114.80 (4)	Ca4—Ca5—As4^i^	94.37 (5)
As3—Ca1—Ca2	50.96 (3)	As1—Ca5—Ca1^i^	56.97 (2)
As1^v^—Ca1—Ca2	118.75 (4)	As1^xi^—Ca5—Ca1^i^	139.54 (6)
As4—Ca1—Ca2	72.78 (3)	As5^i^—Ca5—Ca1^i^	61.07 (3)
As5—Ca1—Ca2	148.16 (4)	As5^x^—Ca5—Ca1^i^	99.68 (4)
Ca6—Ca1—Ca2	70.07 (3)	As4—Ca5—Ca1^i^	128.45 (3)
As1—Ca1—Ca5^v^	117.30 (4)	Ca4—Ca5—Ca1^i^	107.53 (4)
As2—Ca1—Ca5^v^	115.73 (4)	As4^i^—Ca5—Ca1^i^	55.32 (3)
As3—Ca1—Ca5^v^	139.77 (5)	As1—Ca5—Ca1^xvii^	139.54 (6)
As1^v^—Ca1—Ca5^v^	49.73 (3)	As1^xi^—Ca5—Ca1^xvii^	56.97 (2)
As4—Ca1—Ca5^v^	57.39 (3)	As5^i^—Ca5—Ca1^xvii^	99.68 (4)
As5—Ca1—Ca5^v^	50.81 (3)	As5^x^—Ca5—Ca1^xvii^	61.07 (3)
Ca6—Ca1—Ca5^v^	72.00 (4)	As4—Ca5—Ca1^xvii^	128.45 (3)
Ca2—Ca1—Ca5^v^	128.96 (4)	Ca4—Ca5—Ca1^xvii^	107.53 (4)
As1—Ca1—Ca4	51.55 (3)	As4^i^—Ca5—Ca1^xvii^	55.32 (3)
As2—Ca1—Ca4	113.60 (4)	Ca1^i^—Ca5—Ca1^xvii^	102.64 (5)
As3—Ca1—Ca4	146.77 (4)	As1—Ca5—Ca4^x^	110.65 (4)
As1^v^—Ca1—Ca4	116.86 (4)	As1^xi^—Ca5—Ca4^x^	110.65 (4)
As4—Ca1—Ca4	60.17 (3)	As5^i^—Ca5—Ca4^x^	52.32 (3)
As5—Ca1—Ca4	49.97 (3)	As5^x^—Ca5—Ca4^x^	52.32 (3)
Ca6—Ca1—Ca4	113.33 (4)	As4—Ca5—Ca4^x^	52.67 (4)
Ca2—Ca1—Ca4	98.64 (4)	Ca4—Ca5—Ca4^x^	119.60 (5)
Ca5^v^—Ca1—Ca4	67.13 (3)	As4^i^—Ca5—Ca4^x^	146.03 (6)
As1—Ca1—Ca2^v^	133.15 (4)	Ca1^i^—Ca5—Ca4^x^	109.11 (4)
As2—Ca1—Ca2^v^	53.52 (3)	Ca1^xvii^—Ca5—Ca4^x^	109.11 (4)
As3—Ca1—Ca2^v^	110.55 (4)	As1—Ca5—Ca2	55.25 (2)
As1^v^—Ca1—Ca2^v^	54.93 (3)	As1^xi^—Ca5—Ca2	152.12 (6)
As4—Ca1—Ca2^v^	120.16 (4)	As5^i^—Ca5—Ca2	50.42 (2)
As5—Ca1—Ca2^v^	49.54 (2)	As5^x^—Ca5—Ca2	95.09 (4)
Ca6—Ca1—Ca2^v^	105.82 (3)	As4—Ca5—Ca2	70.68 (3)
Ca2—Ca1—Ca2^v^	161.49 (4)	Ca4—Ca5—Ca2	102.03 (3)
Ca5^v^—Ca1—Ca2^v^	62.84 (3)	As4^i^—Ca5—Ca2	115.54 (3)
Ca4—Ca1—Ca2^v^	99.47 (4)	Ca1^i^—Ca5—Ca2	60.28 (3)
As1—Ca1—Ca3^v^	146.01 (4)	Ca1^xvii^—Ca5—Ca2	149.46 (5)
As2—Ca1—Ca3^v^	66.08 (3)	Ca4^x^—Ca5—Ca2	60.47 (3)
As3—Ca1—Ca3^v^	51.14 (3)	As1—Ca5—Ca2^xi^	152.12 (6)
As1^v^—Ca1—Ca3^v^	47.15 (2)	As1^xi^—Ca5—Ca2^xi^	55.25 (2)
As4—Ca1—Ca3^v^	117.83 (4)	As5^i^—Ca5—Ca2^xi^	95.09 (4)
As5—Ca1—Ca3^v^	114.44 (4)	As5^x^—Ca5—Ca2^xi^	50.42 (2)
Ca6—Ca1—Ca3^v^	65.13 (3)	As4—Ca5—Ca2^xi^	70.68 (3)
Ca2—Ca1—Ca3^v^	97.40 (4)	Ca4—Ca5—Ca2^xi^	102.03 (3)
Ca5^v^—Ca1—Ca3^v^	96.35 (4)	As4^i^—Ca5—Ca2^xi^	115.54 (3)
Ca4—Ca1—Ca3^v^	162.04 (5)	Ca1^i^—Ca5—Ca2^xi^	149.46 (5)
Ca2^v^—Ca1—Ca3^v^	65.31 (4)	Ca1^xvii^—Ca5—Ca2^xi^	60.28 (3)
As3—Ca2—As5^i^	173.15 (5)	Ca4^x^—Ca5—Ca2^xi^	60.47 (3)
As3—Ca2—As2^i^	101.80 (3)	Ca2—Ca5—Ca2^xi^	120.73 (5)
As5^i^—Ca2—As2^i^	84.15 (3)	As1^xv^—Ca6—As1^v^	160.05 (7)
As3—Ca2—As2^viii^	97.73 (3)	As1^xv^—Ca6—As4^ix^	92.05 (2)
As5^i^—Ca2—As2^viii^	83.74 (3)	As1^v^—Ca6—As4^ix^	100.50 (3)
As2^i^—Ca2—As2^viii^	47.46 (3)	As1^xv^—Ca6—As4	100.51 (3)
As3—Ca2—As1	91.89 (4)	As1^v^—Ca6—As4	92.05 (2)
As5^i^—Ca2—As1	91.39 (3)	As4^ix^—Ca6—As4	101.95 (5)
As2^i^—Ca2—As1	91.02 (3)	As1^xv^—Ca6—Ca1	115.33 (3)
As2^viii^—Ca2—As1	138.45 (4)	As1^v^—Ca6—Ca1	58.77 (2)
As3—Ca2—As1^xv^	86.41 (3)	As4^ix^—Ca6—Ca1	147.96 (4)
As5^i^—Ca2—As1^xv^	87.09 (3)	As4—Ca6—Ca1	58.76 (2)
As2^i^—Ca2—As1^xv^	131.29 (4)	As1^xv^—Ca6—Ca1^xviii^	58.77 (2)
As2^viii^—Ca2—As1^xv^	84.01 (3)	As1^v^—Ca6—Ca1^xviii^	115.33 (3)
As1—Ca2—As1^xv^	137.10 (4)	As4^ix^—Ca6—Ca1^xviii^	58.76 (2)
As3—Ca2—Ca1	53.93 (3)	As4—Ca6—Ca1^xviii^	147.97 (4)
As5^i^—Ca2—Ca1	124.96 (4)	Ca1—Ca6—Ca1^xviii^	149.70 (6)
As2^i^—Ca2—Ca1	126.48 (4)	As1^xv^—Ca6—Ca4^v^	143.80 (4)
As2^viii^—Ca2—Ca1	151.28 (4)	As1^v^—Ca6—Ca4^v^	50.75 (2)
As1—Ca2—Ca1	50.26 (3)	As4^ix^—Ca6—Ca4^v^	51.76 (3)
As1^xv^—Ca2—Ca1	97.28 (3)	As4—Ca6—Ca4^v^	89.17 (4)
As3—Ca2—Ca2^iv^	50.77 (2)	Ca1—Ca6—Ca4^v^	99.58 (3)
As5^i^—Ca2—Ca2^iv^	133.55 (2)	Ca1^xviii^—Ca6—Ca4^v^	95.54 (3)
As2^i^—Ca2—Ca2^iv^	53.93 (2)	As1^xv^—Ca6—Ca4^x^	50.75 (2)
As2^viii^—Ca2—Ca2^iv^	54.26 (2)	As1^v^—Ca6—Ca4^x^	143.80 (4)
As1—Ca2—Ca2^iv^	106.72 (2)	As4^ix^—Ca6—Ca4^x^	89.17 (4)
As1^xv^—Ca2—Ca2^iv^	105.034 (19)	As4—Ca6—Ca4^x^	51.76 (3)
Ca1—Ca2—Ca2^iv^	98.27 (2)	Ca1—Ca6—Ca4^x^	95.54 (3)
As3—Ca2—Ca1^i^	127.17 (4)	Ca1^xviii^—Ca6—Ca4^x^	99.58 (3)
As5^i^—Ca2—Ca1^i^	59.38 (3)	Ca4^v^—Ca6—Ca4^x^	119.60 (5)
As2^i^—Ca2—Ca1^i^	49.75 (3)	As1^xv^—Ca6—Ca6^ix^	99.97 (3)
As2^viii^—Ca2—Ca1^i^	90.05 (4)	As1^v^—Ca6—Ca6^ix^	99.97 (3)
As1—Ca2—Ca1^i^	53.30 (3)	As4^ix^—Ca6—Ca6^ix^	50.97 (2)
As1^xv^—Ca2—Ca1^i^	146.42 (4)	As4—Ca6—Ca6^ix^	50.97 (2)
Ca1—Ca2—Ca1^i^	103.38 (3)	Ca1—Ca6—Ca6^ix^	105.15 (3)
Ca2^iv^—Ca2—Ca1^i^	97.91 (2)	Ca1^xviii^—Ca6—Ca6^ix^	105.15 (3)
As3—Ca2—Ca1^xv^	117.40 (4)	Ca4^v^—Ca6—Ca6^ix^	59.80 (2)
As5^i^—Ca2—Ca1^xv^	58.75 (3)	Ca4^x^—Ca6—Ca6^ix^	59.80 (2)
As2^i^—Ca2—Ca1^xv^	89.54 (4)	As1^xv^—Ca6—Ca3^viii^	46.20 (3)
As2^viii^—Ca2—Ca1^xv^	49.11 (3)	As1^v^—Ca6—Ca3^viii^	113.85 (5)
As1—Ca2—Ca1^xv^	149.92 (4)	As4^ix^—Ca6—Ca3^viii^	118.74 (3)
As1^xv^—Ca2—Ca1^xv^	46.38 (2)	As4—Ca6—Ca3^viii^	124.43 (3)
Ca1—Ca2—Ca1^xv^	143.14 (4)	Ca1—Ca6—Ca3^viii^	92.94 (4)
Ca2^iv^—Ca2—Ca1^xv^	97.81 (2)	Ca1^xviii^—Ca6—Ca3^viii^	60.95 (3)
Ca1^i^—Ca2—Ca1^xv^	106.95 (4)	Ca4^v^—Ca6—Ca3^viii^	145.51 (4)
As3—Ca2—Ca4^x^	122.20 (4)	Ca4^x^—Ca6—Ca3^viii^	90.66 (3)
As5^i^—Ca2—Ca4^x^	51.17 (3)	Ca6^ix^—Ca6—Ca3^viii^	146.17 (2)
As2^i^—Ca2—Ca4^x^	133.52 (4)	As1^xv^—Ca6—Ca3^v^	113.85 (5)
As2^viii^—Ca2—Ca4^x^	107.46 (4)	As1^v^—Ca6—Ca3^v^	46.20 (3)
As1—Ca2—Ca4^x^	100.95 (4)	As4^ix^—Ca6—Ca3^v^	124.43 (3)
As1^xv^—Ca2—Ca4^x^	47.48 (3)	As4—Ca6—Ca3^v^	118.74 (3)
Ca1—Ca2—Ca4^x^	93.83 (4)	Ca1—Ca6—Ca3^v^	60.95 (3)
Ca2^iv^—Ca2—Ca4^x^	151.41 (3)	Ca1^xviii^—Ca6—Ca3^v^	92.94 (4)
Ca1^i^—Ca2—Ca4^x^	104.36 (4)	Ca4^v^—Ca6—Ca3^v^	90.66 (3)
Ca1^xv^—Ca2—Ca4^x^	58.77 (3)	Ca4^x^—Ca6—Ca3^v^	145.51 (4)
As3—Ca2—Ca3^viii^	51.18 (3)	Ca6^ix^—Ca6—Ca3^v^	146.17 (2)
As5^i^—Ca2—Ca3^viii^	124.16 (4)	Ca3^viii^—Ca6—Ca3^v^	67.66 (5)
As2^i^—Ca2—Ca3^viii^	105.77 (4)	As1^xv^—Ca6—Ca2	59.33 (2)
As2^viii^—Ca2—Ca3^viii^	67.53 (3)	As1^v^—Ca6—Ca2	112.86 (3)
As1—Ca2—Ca3^viii^	141.40 (4)	As4^ix^—Ca6—Ca2	145.04 (3)
As1^xv^—Ca2—Ca3^viii^	44.89 (2)	As4—Ca6—Ca2	67.851 (19)
Ca1—Ca2—Ca3^viii^	93.16 (4)	Ca1—Ca6—Ca2	56.14 (3)
Ca2^iv^—Ca2—Ca3^viii^	61.37 (2)	Ca1^xviii^—Ca6—Ca2	111.93 (3)
Ca1^i^—Ca2—Ca3^viii^	155.47 (4)	Ca4^v^—Ca6—Ca2	152.53 (3)
Ca1^xv^—Ca2—Ca3^viii^	66.39 (3)	Ca4^x^—Ca6—Ca2	57.74 (2)
Ca4^x^—Ca2—Ca3^viii^	92.29 (3)	Ca6^ix^—Ca6—Ca2	110.57 (3)
As1^iv^—Ca3—As1	153.39 (7)	Ca3^viii^—Ca6—Ca2	56.98 (3)
As1^iv^—Ca3—As3^i^	99.68 (3)	Ca3^v^—Ca6—Ca2	87.77 (4)

Symmetry codes: (i) *x*+1/2, −*y*+1/2, *z*; (ii) −*x*+1/2, *y*−1/2, *z*; (iii) −*x*, −*y*, −*z*+1; (iv) *x*, *y*, −*z*+1; (v) *x*−1/2, −*y*+1/2, *z*; (vi) *x*−1/2, −*y*+1/2, −*z*+1; (vii) −*x*+1/2, *y*−1/2, −*z*+1; (viii) −*x*+1/2, *y*+1/2, −*z*+1; (ix) −*x*, −*y*+1, −*z*; (x) −*x*+1/2, *y*+1/2, −*z*; (xi) *x*, *y*, −*z*; (xii) −*x*, −*y*, −*z*; (xiii) −*x*+1/2, *y*−1/2, −*z*; (xiv) −*x*, −*y*, *z*; (xv) −*x*+1/2, *y*+1/2, *z*; (xvi) *x*+1/2, −*y*+1/2, −*z*+1; (xvii) *x*+1/2, −*y*+1/2, −*z*; (xviii) −*x*, −*y*+1, *z*.
